# Modified ground-truthing: an accurate and cost-effective food environment validation method for town and rural areas

**DOI:** 10.1186/s12966-016-0360-3

**Published:** 2016-03-17

**Authors:** Caitlin Eicher Caspi, Robin Friebur

**Affiliations:** Department of Family Medicine and Community Health, University of Minnesota, Program in Health Disparities Research, 717 Delaware St. SE, Minneapolis, MN 55414 USA; Nutrition Policy Institute, University of California Berkeley, 2115 Milvia Street, Suite 3, Berkeley, CA 94704 USA

**Keywords:** Food environment, Validation, Cost-effectiveness, Accuracy, Ground-truthing

## Abstract

**Background:**

A major concern in food environment research is the lack of accuracy in commercial business listings of food stores, which are convenient and commonly used. Accuracy concerns may be particularly pronounced in rural areas. Ground-truthing or on-site verification has been deemed the necessary standard to validate business listings, but researchers perceive this process to be costly and time-consuming. This study calculated the accuracy and cost of ground-truthing three town/rural areas in Minnesota, USA (an area of 564 miles, or 908 km), and simulated a modified validation process to increase efficiency without comprising accuracy. For traditional ground-truthing, all streets in the study area were driven, while the route and geographic coordinates of food stores were recorded.

**Results:**

The process required 1510 miles (2430 km) of driving and 114 staff hours. The ground-truthed list of stores was compared with commercial business listings, which had an average positive predictive value (PPV) of 0.57 and sensitivity of 0.62 across the three sites. Using observations from the field, a modified process was proposed in which only the streets located within central commercial clusters (the 1/8 mile or 200 m buffer around any cluster of 2 stores) would be validated. Modified ground-truthing would have yielded an estimated PPV of 1.00 and sensitivity of 0.95, and would have resulted in a reduction in approximately 88 % of the mileage costs.

**Conclusions:**

We conclude that ground-truthing is necessary in town/rural settings. The modified ground-truthing process, with excellent accuracy at a fraction of the costs, suggests a new standard and warrants further evaluation.

## Background

The community food environment has been widely recognized as a key determinant of dietary behavior and weight outcomes among youth [1–6]. Several studies conducted in urban settings have linked poor quality food environments surrounding schools, such as convenience and other small food stores that sell sugar-sweetened beverages, to adverse diet and weight outcomes [4, 7–9]. Yet, findings have not always been consistent [10–13]. A number of substantial challenges with food environment assessment methodologies make it difficult to critically evaluate the existing body of research, as well as the credibility of their conclusions [3, 14].

In the U.S., the community food environment outside of urban areas (from here on referred to as town/rural areas) has remained understudied, even though these areas have demonstrated a dearth of healthy, high-quality foods [15–17]. Research on town/rural food environments in the U.S. has so far been concentrated in a few pockets [16–21], even while town/rural foodscapes are likely be heterogeneous and context-dependent. A better understanding of the role of town/rural food environments on health is warranted, particularly in light of a recent meta-analysis estimating that the odds of obesity are 26 % higher among youth in rural areas compared with their urban counterparts [22].

A central challenge in conducting food environment research is obtaining accurate food environment data. The most common sources of data listing food outlets are commercial business listings (e.g., Dun & Bradstreet, InfoUSA), which are business marketing tools not meant to be used in health research [23]. These data are convenient and, by some counts, have fueled more than two-thirds of the growing body of research on the food environment and health [24, 25]. Yet, a number of studies call into question the accuracy of these data [20, 21, 23, 26, 27]. Store omissions on these business lists can lead to measurement error [14, 28], resulting in attenuation bias in regression estimates if omissions are random, and more complex bias if errors are more likely in certain store types. Even studies that report reasonable levels of list accuracy acknowledge that accuracy varies by the type of list used [29–31] and by store type [32, 33]. Discrepancies between secondary data listings and actual foodscapes may be particularly pronounced in town/rural area, where geocoding errors are more common [34], stores may close more frequently [21], and “non-traditional” sources of food like dollar stores may be more common [26, 35].

Researchers have increasingly called for ground-truthing as a necessary process to validate business lists [14, 21, 23, 35–37], which involves canvassing all of the streets within a geographic area to enumerate all existing food establishments. Few researchers actually carry this process out, perceiving it as costly and time consuming [38–40]. Of the studies included in a recent systematic review on the retail food environment around schools [11], only 5 out of 28 studies using food store lists reported conducting *any* kind of on-site validation. Few studies have quantified the time and resources needed to ground-truth [35], but Fleischhacker [20] reported that it took 20 data collection days to ground-truth 1502 miles (2417 km). More resource-efficient validation methods could encourage the adoption of higher validation standards among research teams, particularly those conducting community-based research, both in the U.S. and elsewhere.

Developing a method for characterizing the food environment in town/rural settings that is both cost-effective and valid is, therefore, a critical step in conducting rigorous studies that evaluate the link between the food environment and health in understudied and under-resourced settings. Such work is essential in order to address the current lack of evaluable research, quantify the impact of “obesogenic” environments on health outcomes, and identify opportunities for intervention. In order to address some of the barriers related to ground-truthing in the U.S., this study pilot-tested the cost and accuracy of a modified validation method, in order to accurately characterize the food environment in the town/rural setting in Minnesota.

## Methods

### Setting

During the summer of 2014, traditional ground-truthing was conducted in the areas surrounding three high-schools located 60–70 miles (97 – 113 km) outside of the Minnesota Twin Cities metropolitan area (Minneapolis and St. Paul), encompassing 564 miles (908 km) of road. The study area of interest was the food environment surrounding schools, as this study was conducted as part of a larger study examining school breakfast participation in rural Minnesota (Project BreakFAST) [41]. The study area included a road network buffer of 3–5 miles (4.8 – 8 km) surrounding each school, which encompassed the area in which at least 80 % of the students enrolled in the study at the three schools lived. The three schools were selected from a total of 16 schools participating in the intervention study because they represented a range of school characteristics (e.g., school size ranged from 325 to 1605 students, 6 % to 17 % of whom were minority students). Two sites were classified as distant towns and the third site was classified as a remote town by the National Center for Education Statistics (NCES) locale codes.

### Traditional ground –truthing

A list of stores in the study area was obtained from Esri’s Business Analyst (BA), a GIS business analytics system that relies on a list of more than 18 million U.S. businesses from Dun & Bradstreet. North American Industry Classification System (NAICS) retailer codes were used to extract a list of stores from BA that might reasonably sell food. Retailer types included supermarkets, grocery stores, supercenters, convenience stores, gas stations (with or without convenience stores), dollar stores, specialty stores, full-service/limited service restaurants, discount department stores, pharmacies/drug stores, and other miscellaneous retailers (e.g., food/health supplement stores and department stores). Because the focus on food environment features relevant to youth, liquor stores and bars were excluded prior to the analysis (though “bar and grille” restaurants were retained). Also excluded before the analysis were emergency food assistance providers (e.g., food banks) and impermanent retailers (e.g., farmer’s markets).

Road maps for the study area were created before data collection using ArcGIS 10.3. In teams of two, data collectors: 1) drove each street in the study area to identify food retail outlets; 2) logged food outlet geographic coordinates (longitude, latitude); and 3) conducted a “windshield survey” [42, 43] to correspond with each store, including the store outlet name, store type, address, hours open, and a storefront picture. Data collectors entered the store to determine whether the store sold food or beverages where it could not be determined from the store exterior (e.g., some gas-marts or gift shops). In this pilot study, several technology devices were tested while developing the ground-truthing protocol. The first data collection site used a portable Garmin navigational device to manually record waypoint positioning and a camera to capture a storefront image. The second two study areas used a Sky Pro GPS Receiver XGPS160 (a high-sensitivity, Wide Area Augmentation System–enabled GPS unit) paired with GPS Tracks HD (an iPad application connected to the GPS device via Bluetooth) to track and record route, positioning, and waypoints in real time.

At the conclusion of data collection at each site, the ground-truthed track history was compared against BA listings. Four classifications for stores emerged: (1) open/found, (2) new store, (3) not found, (4) ineligible. Stores were classified as open/found if they were found during ground-truthing and matched a BA store name and location. Consistent with a previous protocol [23], matches included *exact matches*, as well as *close matches* (e.g., Mizuki Fusion listed as Zhang Ke Mizuki Fusion), and *lenient matches* where both names suggested a similar vendor type and product line (e.g., Papa Murphy’s instead of Midwest Pizza Group, El Progresso Market instead of Texano Groceries). Addresses were compared to make sure matches were near the same intersection [23]. Stores that were found during ground-truthing that had not appeared on the BA list were classified as new stores. Outlets on the BA list that were not found during ground-truthing (either because they were wrongly listed or because they were no longer present at that location) were classified as not found. Outlets that were found, but should not have been included as food stores, were deemed ineligible. This included exclusive establishments for specific populations, or establishments requiring special membership (e.g., institutionalized settings, cafeterias in hospitals, country clubs) [36] and stores that, upon visiting, were confirmed not to sell food.

### Measures

Stores that were open/found were considered to be “true positives.” New stores were considered to be “false negatives.” Those not found, closed, or ineligible were all considered to be “false positives,” as these stores would likely have been erroneously assumed to be present, open, and relevant to the food environment if no validation had been done.

The positive predictive value (PPV) of the BA list was the probability that stores were located and open where they were listed on the BA list, calculated as true positives/(true positives + false positives). Sensitivity was the probability that open stores were listed on the BA list, calculated as true positives/(true positives + false negatives).

Cost metrics included two mileage measures: (1) ground-truthing mileage (2) total mileage (including traveling to and from the sites). Mileage costs were estimated at $0.565 per mile, the mileage reimbursement rate at the institution where the research took place. Cost metrics also included time (hours spent ground-truthing, miles per hour during ground-truthing, and total number of hours of field work for two data collectors). At the first site, mileage and time estimates were calculated from the car odometer and standard car clock; in the other sites, hours and miles per hour were automatically monitored by the HD Tracks app; tracking was paused during breaks.

### Modified ground-truthing

While conducting ground-truthing, the researchers made a number of observations about the spatial patterning of store locations. They noted that nearly all stores were concentrated in a small number of commercial clusters; that stores listed outside these areas were likely to be false positives; and that most of the data collection time was spent driving through areas where there were no retailers (e.g., unpaved back roads, long stretches of remote country roads, pockets of housing developments with *cul de sacs*, and residential grids in town centers). This led to a hypothesis that a more efficient ground-truthing protocol could be developed for town/rural areas with minimal compromises in accuracy.

To test this hypothesis, a modified protocol was proposed. According to this protocol, data collectors would use the original BA lists to identify key, targeted areas (central commercial clusters) that were likely to yield the most information for validation. ArcGIS was used to simulate the accuracy (PPV and sensitivity) of this process by locating the ground-truthed stores that fell inside and outside central commercial clusters, defined in this study as the 1/8 mile (200 m) buffer around any cluster of at least two stores. Accuracy results (PPV and sensitivity) were recalculated, comparing the list of stores that would have been generated if only the central commercial areas had been ground-truthed. Two scenarios were considered: (1) a list comparison that assumed all stores outside the central commercial clusters were open; and (2) a list comparison that assumed all stores outside the central commercial clusters were closed.

Potential cost-savings were estimated for mileage and number of trips taken. It was not possible to estimate time savings since the rate of data collection (miles per hour) in central commercial clusters was likely substantially different from the rate in areas where there were no retailers.

## Results

Accuracy results are presented in Table 1. Findings showed that the PPV for the BA list ranged from 0.50 to 0.65 across the three sites (average 0.57). Sensitivity ranged from 0.50 to 0.70 (average 0.62). Out of 136 stores identified, 34 % were not on the BA list. Furthermore, 45 % of the stores on the BA list should not have been on the list because they were not found (*n* = 45) or ineligible (*n* = 16).

**Table 1:** Accuracy of three food retail outlet lists, as compared with ground-truthing

		List 1: Business Analyst (BA)				List 2: Modified ground-truthing,^a^ (stores outside of clusters assumed *open)*				List 3: Modified ground truthing,^a^ (stores outside of clusters assumed *closed)*			
		Site				Site				Site			
		A	B	C	Total	A	B	C	Total	A	B	C	Total
Stores on list	for comparison with traditional ground-truthing	14	74	63	151	15	78	60	153	14	66	46	126
True positives	stores correctly listed	7	48	35	90	14	69	49	132	14	66	46	126
False positives	stores that should not have been on the list	7	26	28	61	1	9	11	21	0	0	0	0
False negatives	stores missing from the list	7	24	15	46	0	3	1	4	0	6	4	10
					Mean				Mean				Mean
Positive Predictive Value (PPV)	true positives/(true positives + false positives)	0.50	0.65	0.56	0.57	0.93	0.88	0.82	0.88	1.00	1.00	1.00	1.00
Sensitivity	true positives/(true positives + false negatives)	0.50	0.67	0.70	0.62	1.00	0.96	0.98	0.98	1.00	0.92	0.92	0.95

^a^The list of stores derived from driving only the streets within 1/8 mile buffer of any two stores on the commercial list

Under the modified ground-truthing scenario, when it was assumed that stores outside the targeted observation area were open, the PPV ranged from 0.82 to 0.93 (average 0.88) and the sensitivity was 0.96 to 1.00 (average 0.98). In this scenario, only 4 new stores (out of a total of 46) would have been missed. However, 21 false positives would still have been included on the list (out of a total of 61). When it was assumed that stores outside the observation area were closed, the PPV was perfect in all three sites (1.00) because there were no false positives. The sensitivity ranged from 0.92 to 1.00 (average 0.95).

Findings also showed that the ground-truthing process was time-intensive (Table 2). Six full days (averaging 9.5 h each) for two staff were needed to ground-truth 564 miles (908 km) of road. Ground-truthing also had additional “hidden” mileage costs. Back-tracking was required to reach all corners of the buffer zone, and these added 27 %, on average, to the mileage. Additionally, data collection required travel to and from the sites; when data could not be completed in one day, additional mileage to and from the site was accrued. Including back-tracking and to-from transit, 1510 total miles (2430 km) were driven to ground-truth 564 miles (908 km) of road.

**Table 2:** Actual costs of traditional ground-truthing compared with estimated costs of modified ground-truthing

	Traditional ground-truthing (actual)				Modified ground-truthing (estimated)^b^				Savings^b^	
	Site				Site					
	A	B	C	Total	A	B	C	Total	Amount	%
Mileage										
Miles of road within network	80	236	248	564	10	32	25	66	498 miles	88 %
Miles driven for ground-truthing	100	262	356	718	12	40	31	83	635 miles	88 %
Ground-truthing mileage costs^a^	$57	$148	$201	406	$7	$23	$18	47	$359	88 %
Miles driven to/from sites	124	272	396	792	124	136	132	392	400 miles	51 %
Total miles driven	224	534	752	1510	136	168	157	460	1050 miles	70 %
Total mileage costs^a^	$127	$302	$425	853	$77	$95	$89	260	$593	70 %
Time/staffing										
Full day trips needed	1	2	3	6	1	1	1	3	3 days	50 %
Time spent ground-truthing (hr:min)	4:30	13:14	15:39	33:38	--	--	--	--	--	--
Average mph during ground truthing	22.2	19.8	22.7	21.4	--	--	--	--	--	--
Total hours of data collection^c^	15	40	59	114	--	--	--	--	--	--

^a^Assuming a mileage rate of $0.565

^b^Not all time/staffing and savings metrics could be estimated. Miles per hour within central commercial districts is likely lower than the miles per hour in all town/rural areas

^c^Hours totaled for two data collectors

Modified ground-truthing would have resulted in a road-network distance of 66 miles (106 km) to be ground-truthed (a savings of 88 %). The process would have required only one visit per site (a 50 % savings in data collection trips). Assuming a similar amount of back-tracking as in traditional gound-truthing (an extra 27 %), the modified process would have resulted in an estimated total of 460 miles (740 km) driven (a 70 % reduction in total miles) compared with traditional ground-truthing.

## Discussion

Conducting traditional ground-truthing in three town/rural sites revealed that on-site validation is, indeed, necessary for accurate analyses of food retail environments. The process also revealed that the current gold-standard of ground-truthing methods is not an efficient method of validation. The positive predictive value and sensitivity observed in the current study are similar or slightly lower than those previously reported in similar studies [20, 27, 29, 36]. For instance, one study reported that the PPV for all stores outside of urban areas using Dun & Bradstreet ranged from 0.67 to 0.78, while the sensitivity fell in the range on 0.54 to 0.65 [26]. Yet, despite the necessity of validation, the equivalent of nearly three weeks of full-time work (114 total staff hours) were needed to ground-truth a relatively small study area and identify 46 new stores.

Additionally, systematic canvassing is tedious work, particularly in areas where stores are few and far between. Streamlining the process without compromising accuracy would allow researchers to place much-needed resources into other components of the research project. This type of streamlining would also make the validation process feasible for community-based organizations conducting assessments on community food environments, and could even make use of citizen science for collecting or verifying data in very remote areas.

A modified ground-truthing process in which only the central commercial clusters were validated would have resulted in substantial time and monetary savings. Results estimated an 88 % reduction in the total number of roads to validate and mileage costs, as well as a 50 % reduction in data collection trips. Further, this simulation suggested that the modified process would have resulted in only very modest compromises in accuracy. These savings would be possible because so few stores were actually found outside of central commercial zones in rural areas. Our results demonstrated that assuming that stores not directly observed within clusters were closed offered the best overall best accuracy. This process demonstrated perfect PPV and only a small compromise in sensitivity (0.95).

Other validation techniques, such as remote-sensing (e.g., Google Street View), have also been touted as cost-effective validation tools [38, 40]. In urban areas, the reliability of such tools has been variable [32, 37, 39, 44]. Outside urban areas, however, use of these methods may present particular challenges [45]. Not all streets can be visualized via remote-sensing [46], and store closings are more common in rural areas [21], meaning that images may not be current. Date stamps are becoming more common on remote sensing images in the U.S., but images still may be out of date or misaligned with the health data to which it is being linked [47]. Visualizing shopping centers (which are often a cornerstone of commerce outside of urban areas and often have a haphazard spatial arrangement) may also be problematic due to image disruption and lack of image continuity [48]. Until issues with remote sensing can be resolved and evidence of their accuracy in rural areas can be demonstrated, modified ground-truthing offers promise as a cost-effective and valid method for creating accurate exposure metrics of the food environment. In conducting food environment research based on business lists, one cost that remains constant across validation methods (traditional ground-truthing, modified ground-truthing, remote sensing or no ground-truthing) is the cost of obtaining business listings. Commercial databases like Dun & Bradstreet are one choice for business lists, and may be licensed to some institutions at a relatively affordable price, but it should be noted that other economical options for data acquisition exist. For instance, administrative data on licensed food outlets may be obtained for free from government agencies (e.g., local health departments or state agriculture departments), and in some cases may be the most reliable [20, 29]. Reliable food environment metrics are needed to accurately estimate the relationship between the food environment and dietary behaviors and health outcomes. In the current body of literature, an abundance of mixed findings on the food environment-diet relationship [3, 11, 23, 24] have led to excessive replication of studies with flawed exposure measures. As a result, the current literature offers few clear conclusions that can be translated into evidence-based policy or interventions for improving nutrition environments in rural areas.

While promising, the modified ground truthing procedure tested in this study requires further exploration. Next steps for research might include testing the appropriateness of this protocol in both more rural and more urban areas. In the most remote rural areas, a larger buffer distance for ground-truthing might be required. In urban areas, the value of using modified ground-truthing might depend on the spatial arrangement of urban food retailers. For instance, modified ground-truthing might be less cost-effective in urban areas with a dense, regular patterning of stores (e.g., New York City [23]), but more cost-effective in areas where stores tend to cluster in certain areas (e.g.,cities with greater sprawl, suburban areas). Once ground-truthing protocols are established, an important next step might be to test the feasibility of adding a brief checklist that reflects store healthy food availability in the modified ground-truthing process. Currently, researchers often designate food retailers as “unhealthy” or “healthy” based solely on their store type, without regard to what they actually sell [14, 23, 24, 35, 49, 50]. As long as data collectors are visiting stores, adding a modified NEMS-S and NEMS-R with just 9 or 16 items [51] might be one way to gather a contained amount of information that could be used to create more nuanced geographic exposure measures, although the added time required to do this would need to be evaluated.

### Limitations

The results of this pilot study should be considered within the study limitations. First, the results of the modified ground-truthing process use simulations only; actually conducting modified ground-truthing could determine whether there were unanticipated costs or challenges associated with the method. For instance, if central commercial areas were geographically dispersed, costs would include substantial unforeseen mileage. Another limitation was that, given the small study area and sample size, we did not report the PPV and sensitivity by store type, even though previous studies have indicated differences by store type [23, 26–28]. Next, this was a small pilot study conducted in one region of Minnesota and represents a small geographic area. As such, store geography may not be representative of more remote rural areas, or of town/rural areas in other parts of the country or other countries. Despite limited generalizability, modified ground-truthing is a practical idea that could be adapted to other regions, both within the U.S. and outside, with a relatively simple assessment of local store geography – for instance, widening the buffer distance if stores are more dispersed. Additionally, it should be noted that ground-truthing is only useful for generating food environment variables that measure residents’ *potential* exposure to food outlets [35]; *actual, realized* exposure of the food environment, which might be measured by GPS tracking or wearable cameras [52], might be more directly relevant to behaviors and health outcomes. While acknowledging this as a limitation, we also wish to recognize that the broader study goal was to advance methods for determining spatial exposures, given that researchers do not always have the resources for detailed tracking of individuals, and reliance on spatial measures shows no signs of slowing.

Finally, one of the limitations of ground-truthing is that it can only capture the present environment. Some of the mismatch between business lists and ground-truthed lists is likely due to temporality – for instance, stores that were once open, but closed before the researchers visited. Temporality is, therefore, a component of validity that must be considered, especially when linking food environment measures to health measures. When linking older health measures to the food environment, ground-truthing may yield inaccurate exposure measures due to temporal mismatch. Ideally, food environments should be validated as close as possible to the time that health measures are collected.

## Conclusions

Taken together with other literature, results from this study of three town/rural areas in Minnesota indicate that an on-site validation process is, indeed, a necessary step in avoiding list errors when conducting community food environment research. Excellent accuracy can be achieved through careful selection of key areas to focus validation efforts, indicating that a modified process could become a new standard for validation. It is unclear to what extent criteria for validating stores may vary in different types of town/rural settings. Given the current reliance on commercial business listings in public health research, such exploration would be a worthwhile investment, particularly for research conducted in low-resource community settings.
